# Potential Ago2/miR-3068-5p Cascades in the Nucleus Accumbens Contribute to Methamphetamine-Induced Locomotor Sensitization of Mice

**DOI:** 10.3389/fphar.2021.708034

**Published:** 2021-08-13

**Authors:** Dan Liu, Min Liang, Li Zhu, Ting-ting Zhou, Yu Wang, Rui Wang, Fei-fei Wu, Eyleen L. K. Goh, Teng Chen

**Affiliations:** ^1^ College of Forensic Medicine, Xi’an Jiaotong University Health Science Center, Xi'an, China; ^2^ The Key Laboratory of Health Ministry for Forensic Science, Xi’an Jiaotong University, Xi'an, China; ^3^ Guangdong Provincial Key Laboratory of Brain Connectome and Behavior, CAS Key Laboratory of Brain Connectome and Manipulation, The Brain Cognition and Brain Disease Institute, Shenzhen-Hong Kong Institute of Brain Science-Shenzhen Fundamental Research Institutions, Shenzhen Institute of Advanced Technology, Chinese Academy of Sciences, Shenzhen, China; ^4^ Department of Research, National Neuroscience Institute, Singapore, Singapore; ^5^ Neuroscience and Mental Health Faculty, Lee Kong China School of Medicine, Nanyang Technological University, Singapore, Singapore

**Keywords:** Ago2, Grin1, locomotor sensitization, methamphetamine, miR-3068-5p

## Abstract

Dysregulation of microRNA (miRNA) biogenesis is involved in drug addiction. Argonaute2 (Ago2), a specific splicing protein involved in the generation of miRNA, was found to be dysregulated in the nucleus accumbens (NAc) of methamphetamine (METH)-sensitized mice in our previous study. Here, we determined whether Ago2 in the NAc regulates METH sensitization in mice and identified Ago2-dependent miRNAs involved in this process. We found a gradual reduction in Ago2 expression in the NAc following repeated METH use. METH-induced hyperlocomotor activity in mice was strengthened by knocking down NAc neuronal levels of Ago2 but reduced by overexpressing Ago2 in NAc neurons. Surprisingly, miR-3068-5p was upregulated following overexpression of Ago2 and downregulated by silencing Ago2 in the NAc. Knocking down miR-3068-5p, serving as an Ago2-dependent miRNA, strengthened the METH sensitization responses in mice. These findings demonstrated that dysregulated Ago2 in neurons in the NAc is capable of regulating METH sensitization and suggested a potential role of Ago2-dependent miR-3068-5p in METH sensitization.

## Introduction

Methamphetamine (METH) is a widely abused psychoanaleptic that induces cognitive impairment or psychotic episodes in mammals (Ikeda et al., 2013; Zhong et al., 2016; Greening et al., 2019) and may cause a large number of serious social criminal issues. METH is a functional dopamine agonist that induces locomotor sensitization by producing dysfunctional mesolimbic dopaminergic systems, including the nucleus accumbens (NAc) (Buchanan et al., 2010; Mizoguchi and Yamada, 2019). Locomotor sensitization, which reflects motivation and psychosis (Chen et al., 2014), heightens the sensitivity of behavioral effects in response to repeated intermittent psychostimulant exposure to the same or lower dose (Robinson and Becker, 1986). This locomotor sensitization in response to METH is long-lasting, indicating that alterations in molecule and gene expression occur in relevant brain regions, such as the NAc (Nestler and Malenka, 2004; Jayanthi et al., 2014). Sustained effort has been devoted to determining the mechanisms of METH-induced locomotor sensitization (METH sensitization) to find a more precise target to cure METH addiction.

MicroRNAs (miRNAs) are among the multiple factors underlying the dynamic adjustment of gene expression at the posttranscriptional level (Fabian et al., 2010; Störchel et al., 2015). miRNAs represent an important class of noncoding RNAs that can inhibit mRNA translation and accelerate their decay by binding to their 3′-untranslated regions (3′ UTR) (Bartel, 2004). In mammals, the primary transcripts of miRNAs undergo endonucleolytic processing by Drosha/Dgcr8 in the nucleus to generate precursors of miRNAs (pre-miRNAs), which are then exported to the cytoplasm to be spliced into approximately 22-nucleotide (nt) mature miRNAs by Dicer1 (Chendrimada et al., 2005). Then, the miRNAs are loaded into the RNA-induced silencing complex (RISC) by association with the Argonaute2 (Ago2) protein, which is responsible for silencing target mRNAs by mRNA degradation or repressing translation (Chendrimada et al., 2005). miRNAs are capable of regulating neuronal development, spine morphogenesis, and synaptic function (Fabian et al., 2010; Störchel et al., 2015). Thus, it is not surprising that dysregulation of miRNAs and their biogenesis is involved in several neurological and neuropsychiatric diseases, such as ALS and drug addiction (Schaefer et al., 2010; Emde et al., 2015; Störchel et al., 2015). Therefore, miRNAs function in different areas and cell types of the brain as members of physiological and disease states, and they could potentially be used as medicines because of their selectivity and small size, allowing them to penetrate the blood-brain barrier.

The Ago2 protein is essential for miRNA-mediated gene silencing and has endonuclease activity for splicing pre-miRNA to miRNA (Ha and Kim, 2014). Furthermore, the splicing function of Ago2 is selective, and only a fraction of miRNAs can be spliced by Ago2. For example, maturation of miR-451, which is important for erythropoiesis, requires Ago2 but not Dicer1 (Cheloufi et al., 2010). The roles of Ago2 in mouse brain development, neurodegenerative diseases, dendritic spine plasticity, and addiction have been studied (Juvvuna et al., 2012; Garcia-Perez et al., 2013; Pircs et al., 2018; Rajgor et al., 2018; Liu et al., 2019). Ago2 deficiency in dopamine 2 receptor (DRD2)- expressing neurons reduced the motivation for cocaine self-administration in mice by dysregulating miRNAs (Schaefer et al., 2010; Shekar et al., 2011). Overexpression or enhanced activity of Ago2 elicited specific changes in miRNAs and mRNAs and showed a strong relationship with high-risk myeloma (Zhou et al., 2010; Hagiwara et al., 2012; Zhang et al., 2013). In our previous study, we found a set of downregulated miRNAs and decreased levels of Ago2 in response to METH (Liu et al., 2019). Therefore, a better understanding of how Ago2 regulates METH sensitization and identifying Ago-dependent miRNAs in METH sensitization would provide new insights into METH addiction.

Here, we found that Ago2 was downregulated progressively in the NAc of mice following METH administration. Adeno-associated virus (AAV)-mediated neuron-specific overexpression of Ago2 (AAV-SYN-Ago2) in the NAc attenuated METH sensitization (20%), while knocking down the NAc neuronal levels of Ago2 (AAV-SYN-shAgo2) enhanced the effect of METH. We further identified an Ago2-dependent miRNA, miR-3068-5p, that was upregulated or downregulated when Ago2 was overexpressed or knocked down in the NAc, respectively. Consistent with this, AAV-mediated neuron-specific knockdown of miR-3068-5p also enhanced METH sensitization and caused induction of *Grin1*, an N-methyl-D-aspartate receptor (NMDAR) subunit that plays a role in the plasticity of synapses (Chiu et al., 2018). Our results demonstrated that neuron-specific expression of Ago2 in the NAc plays a role in regulating METH sensitization. We further identified Ago2-dependent miR-3068-5p as part of a potential mechanistic cascade regulating METH sensitization.

## Materials and Methods

### Animals

Eight-to-ten-week-old wild-type C57BL/6J mice (Beijing Vital River Laboratory Animal Technology, Beijing, China) weighing 25–30 g were used in this research. Mice were housed four per cage in a temperature-controlled (21–25°C) and humidity-controlled (40–60%) room with a 12 h light/dark cycle (lights on from 7:00 to 19:00) and ad libitum access to chow and water. All behavioral tests were conducted during the light cycle. Mice were habituated to these housing conditions for 7 days and handled daily before starting the experiments. Animal procedures were conducted in accordance with the United Kingdom Animals (Scientific Procedures) Act and Institutional Animal Care Committee at Xi’an Jiaotong University.

### Drugs

METH hydrochloride (National Institute for the Control of Pharmaceutical and Biological Products, Beijing, China) was dissolved in 0.9% physiological saline to a concentration of 0.2 mg/ml for injections. The dose of METH used here was 2 mg/kg, which was injected intraperitoneally (i.p.) at a volume of 10 ml/kg.

### Adeno-Associated Virus

Neural AAV expressing synapsin-1 (SYN) specific promoters was supplied by OBiO Technology (Shanghai, China). AAV-SYN-Ago2 was used to mediate overexpression of Ago2 (NM_153178); AAV-SYN-shAgo2 was used to mediate shRNA expression to interfere with Ago2; AAV-SYN-spmiR-3068-5p was used to mediate “miRNA sponge” expression to inhibit miR-3068-5p; AAV-SYN-spmiR-30a-5p was used to mediate “miRNA sponge” expression to inhibit miR-30a-5p. The final preparation was titrated by quantitative real-time PCR (qPCR), and all titers of the viral vector were over 2.5E+12 vg/ml. AAV was expressed for 4 weeks, and a behavioral test was performed.

### Stereotaxic Surgery

Mice were anesthetized using isoflurane and positioned onto a stereotaxic apparatus (RWD, Shenzhen, China). AAVs were injected bilaterally into the NAc (0.4–0.6 μl per side, 0.2 μl/min, AP: +0.16 cm from bregma, ML: ± 0.26 cm from the midline, and DV: −0.48 cm from the skull at 20° angle) (Franklin and Paxinos, 2001; Aguilar-Valles et al., 2014) with a Hamilton microsyringe (Hamilton 1700 series, Nevada, United States) and an automated injection pump (RWD, Shenzhen, China). After the infusion was completed, the microsyringe was left in place for 6 min to allow for diffusion of AAV complexes. Mice were housed with free access to food and water and given standard care. Four weeks after AAV microinjection, the targeted sites were verified by examining GFP *via* fluorescence microscopy (Leica DM3000, Oskar, Germany), and the up- or downregulation of each molecule was detected by qPCR or Western blot (WB).

### Locomotor Activity Test

**FIGURE 1:**
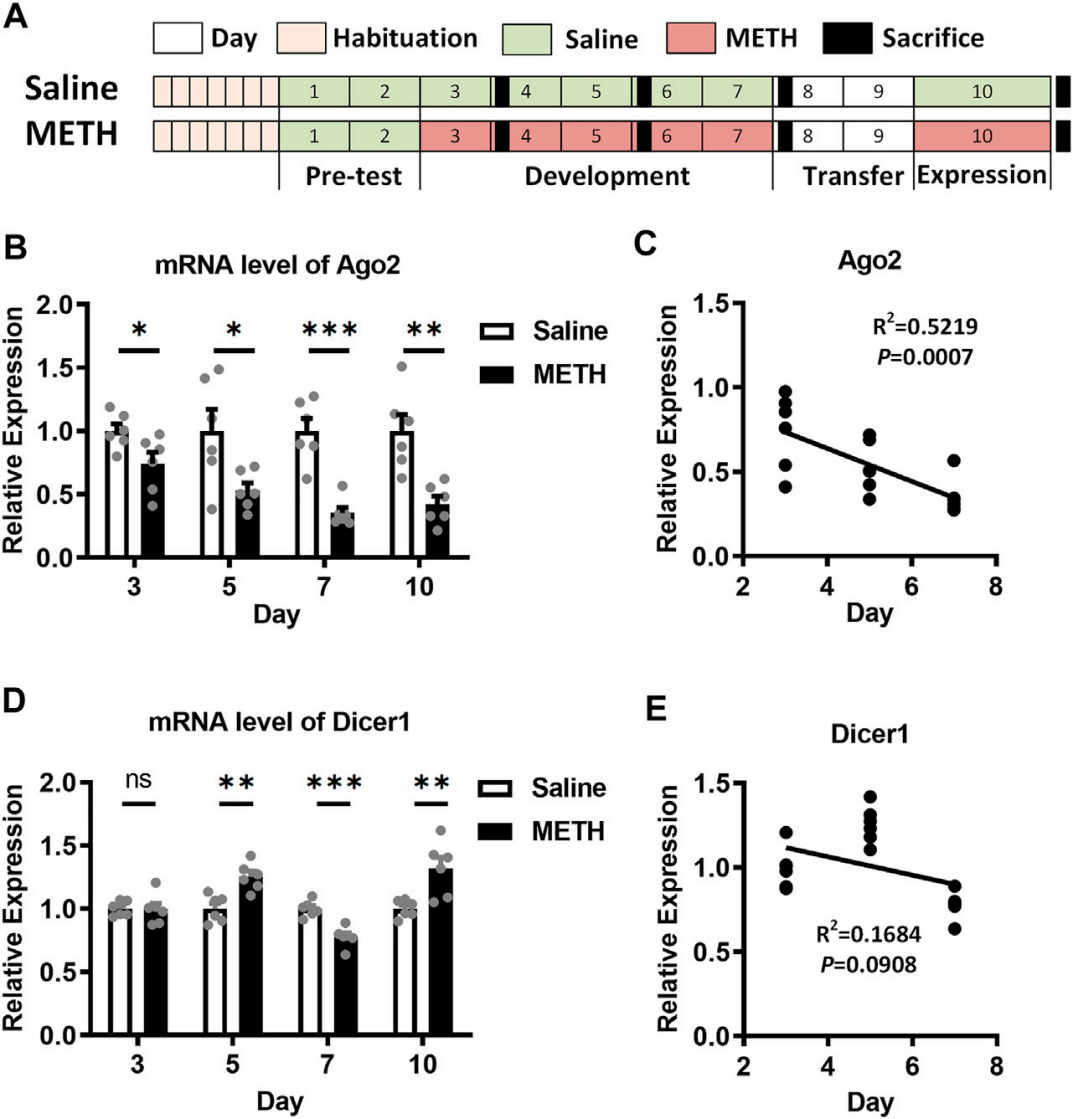
Expression of Ago2 and Dicer1 in the NAc of METH-sensitized mice. **(A)** Illustrations showing the schedule and procedure of the METH sensitization model. **(B)** Differential expression of Ago2 mRNA in the NAc of mice in response to METH. **(C)** Pearson’s correlation analyses between Ago2 expression and days in the development phase of METH sensitization. **(D)** Differential expression of Dicer1 mRNA in the NAc of mice in response to METH. **(E)** Pearson’s correlation analyses between Dicer1 expression and days in the development phase of METH sensitization. Student’s *t*-test: *p < 0.05, **p < 0.01, and ***p < 0.001 compared with the saline group. The data are presented as the mean ± SEM, *n* = 6. NAc, nucleus accumbens; METH sensitization, METH-induced locomotor sensitization.

METH-induced locomotor sensitization was quantified using an open-field (OF) test (Figure 1A (Liu et al., 2019)). After 7 days of habituation, the experiments were initiated with 2 days of saline injection (days 1–2, pretest). Mice were then randomly allocated into the saline or METH treatment groups. The METH or saline group was treated with METH or saline for 5 consecutive days (day 3–7, the development phase). Subsequently, the same doses of the METH or saline challenge injections (day 10, the expression phase) were given after an injection-free interval of 2 days (days 8–9, the transfer phase). Horizontal locomotor activities were recorded in metal test chambers (43 cm × 43 cm × 43 cm) and analyzed for 60 min after injection using a smart video tracking system (version 2.5; PanLab Technology for Bioresearch, Barcelona, Spain).

### Tissue Preparation

Mice were sacrificed 24 h after the last injection, and their brains were rapidly removed. The NAc (+ 1.70 mm from bregma (Franklin and Paxinos, 2001), including the core and shell, was identified based on structure and landmarks under a dissecting microscope and was separated bilaterally. The whole NAc was then immediately frozen in liquid nitrogen.

For RNA extraction, total RNA was isolated by the miRNeasy Mini Kit (217004, Qiagen, United States). The RNA concentration and quality were determined with a NanoDrop spectrophotometer (Thermo Scientific, United States). For miRNA reverse transcription, 380 ng of total RNA per sample was reverse-transcribed to 10 μl of cDNA with the Mir-X™ miRNA First-Strand Synthesis Kit (Takara Biomedical Technology, Beijing, China) at 37°C for 60 min and 85°C for 5 s. cDNA samples were stored at −80°C for further use. For mRNA, 500 ng of total RNA was reverse-transcribed into 10 μl of cDNA with Prime Script ™ RT Master Mix (Takara Biomedical Technology, Beijing, China) by incubating at 37°C for 15 min, 85°C for 5 s, and 4°C for 5 min.

For protein extraction, NAc tissues were homogenized in RIPA (HEART WB009, Xi’an, China) lysis buffer with proteinase and a phosphatase inhibitor (Roche, Shanghai, China). After 60 min of incubation on ice, the homogenates were centrifuged at 12,000 ×g for 5 min at 4°C. The supernatants were collected, and the protein concentrations were measured using the Bradford BCA protein assay (Applygen Technologies Inc. P1511, Beijing, China). Protein homogenates were stored at −80°C for further use.

### Quantitative Real-Time Reverse Transcription PCR

qPCR for miRNA detection was performed with SYBR Premix Ex Taq II (Takara Biomedical Technology, Beijing) using a Bio-Rad iQ5 detection instrument (Bio-Rad, United States) under the following conditions: 95°C for 30 s, followed by 40 cycles of 95°C for 10 s and 62°C for 60 s. *U6* snRNA was used as an endogenous control for detecting miRNAs, and *Gapdh* was the endogenous control for measuring protein-coding gene expression. The relative expression levels were determined using the 2^−△△Ct^ method (Schmittgen and Livak, 2008). miRNAs were then ligated to 3′ adaptors and reverse-transcribed to cDNAs in step extraction. A uni-miR qPCR primer (Takara Biomedical Technology, Beijing) was used as the reverse primer, and the mature miRNA sequences were used as forward primers. The sequences of the primer pairs for protein-coding genes are shown in Table 1.

**TABLE 1:** qPCR primers.

Gene	Forward (5′-3′)	Reverse (5′-3′)
*Gapdh*	TGT​GTC​CGT​CGT​GGA​TCT​GA	TTG​CTG​TTG​AAG​TCG​CAG​GAG
*Dicer1*	GAA​TTG​CTC​GAG​ATG​GAA​CCA​GA	AGC​TCC​GGC​CAA​CAC​CTT​TA
*Ago2*	ACATTCCCGCAGGCACAA	GTC​ATC​CCA​AAG​CAC​GTG​GTA​G
*Grin1*	GGC​TGA​CTA​CCC​GAA​TGT​CCA	TGT​AGA​CGC​GCA​TCA​TCT​CAA​AC
*Gabbr1*	ACG​TCA​CCT​CGG​AAG​GTT​G	CAC​AGG​CAG​GAA​ATT​GAT​GGC
*Msfd2a*	AAC​AAG​CTT​TGC​TAT​GCA​GTT​GGA​G	GCT​AAT​GCA​GAA​GCC​CAC​CAG
*Agt*	GGG​TCA​GTA​CAG​ACA​GCA​CCC​TA	CGG​AGA​TCA​TGG​GCA​CAG​AC
*App*	TTC​TGG​GCT​GAC​AAA​CAT​CAA​GAC	GGT​GAT​GAC​AAT​CAC​GGT​TGC​TA

### Western Blot

Protein homogenates were prepared with 5× protein loading buffer (HEART R0891, Xi’an, China) and denatured at 95°C for 5 min. Fifteen micrograms of protein per sample was resolved on a precast 10% (w/v) SDS-PAGE gel and transferred onto a polyvinylidene fluoride (PVDF) membrane (Millipore IPVH00010, Bedford, MA, United States). Blots were blocked with 5% (w/v) nonfat milk solution (in Tris-buffered saline with 0.1% Tween-20 (TBST)) and then incubated overnight at 4°C in primary antibody solutions (Anti-Ago2, Abcam, ab186733, diluted 1:2000). Membranes were then washed with TBST and probed with the appropriate horseradish peroxidase-conjugated secondary antibodies (1:2000) for 1 h at room temperature. Membranes were visualized using an enhanced chemiluminescence detection kit (Solarbio PE0010, Beijing, China) and quantified with ImageLab 1.46 (BioRad, United States).

### Ingenuity Pathway Analysis Bioinformatics Analysis

Ingenuity Pathway Analysis (IPA) software (version 2019 summer) (Ingenuity Systems, Redwood City, CA, United States; apps.ingenuity.com) was used to characterize the molecular function and regulatory mechanism together with the differentially expressed mRNAs that were identified previously by Zhu et al. with miR-3068-5p target genes predicted by TargetScan (http://www.targetscan.org/vert_71/). Annotation of biological diseases and functions and identification of interaction networks were conducted.

### Dual-Luciferase Reporter Assay

The 3′UTR of *Grin1* (*Grin1* 3′UTR (Wt)) was cloned into the pMIR vector with the firefly luciferase coding region (OBiO Technology, Shanghai, China). The *Grin1* 3′UTR (Mu) was derived from the *Grin1* 3′UTR (Wt) by mutating the miR-3068-5p seed site. 293T cells were inoculated into 96-well plates. The luciferase reporter vector DNA and mimic-miR-3068-5p (OBiO Technology, Shanghai, China) were cotransfected into 293T cells. The relative luciferase activity of 293T cells was assayed by the Dual-Luciferase^®^ Reporter Assay (Spark 10M, TECAN). pRL-CMV containing Renilla luciferase was cotransfected with the 3′UTR of *Grin1* for data normalization, and the data are expressed as Luc/R-luc.

### Statistical Analysis

Statistical analyses were performed using SPSS 18.0 or Prism 6. For the OF test, mixed-measures ANOVA and then multiple comparisons tests were performed to determine significance for the 8 days of the OF test, with days as the within-subject variable and treatments (AAV and METH) as the between-subject factor. qPCR data were standardized by the 2^−△△Ct^ method with *Gapdh/U6*, and Student's *t*-test or two-way ANOVA (Tukey's multiple comparisons test) was used to analyze the expression changes. For Western blots, data normalized to β-actin were analyzed by Student's *t*-test. The data are expressed as mean ± SEM. *p*-values < 0.05 were defined as significant.

## Results

### Progressive Downregulation of Argonaute2 in Response to Methamphetamine

In our previous study, both the Ago2 mRNA and protein were found to be downregulated in the NAc of METH-sensitized mice (Liu et al., 2019). To further investigate the role of Ago2 in METH sensitization, we measured Ago2 mRNA expression in the NAc of mice during the development and expression phases of METH sensitization. Ago2 in METH-treated mice showed progressively downregulated expression, where 26% (t_(10)_ = 2.427, *p < 0.05), 47% (t_(10)_ = 2.587, *p < 0.05), 65% (t_(10)_ = 5.900, ***p < 0.001), and 58% (t_(10)_ = 4.054, **p < 0.01) decreases relative to the control were detected 24 h after injection at days 3, 5, 7, and 10 (Figure 1B). Furthermore, the mRNA level of Ago2 was significantly negatively correlated (F_(1, 16)_ = 17.46, *p < 0.05) with the day of the development phase of METH sensitization (Figure 1C). Another miRNA biogenesis enzyme, Dicer1, also showed stochastic changes during the timeline of our model (Figure 1D). However, there was no correlation (F_(1, 16)_ = 3.239, *p* > 0.05) between the mRNA level of Dicer1 and the development phase of METH sensitization (Figure 1E).

### Methamphetamine Sensitization Can Be Regulated by Argonaute2 in Nucleus Accumbens Neurons

**FIGURE 2:**
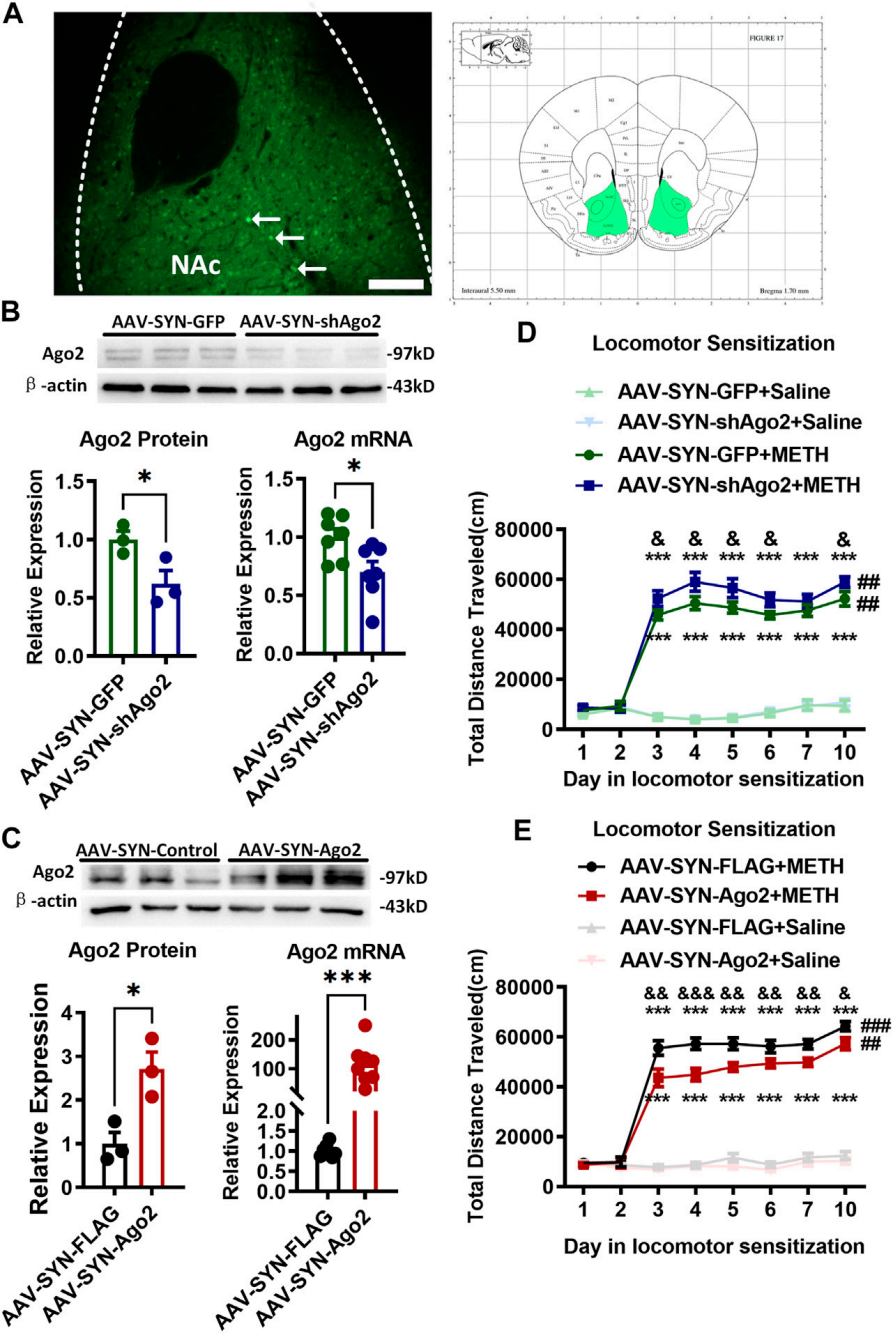
Regulation of Ago2 in NAc neurons affected METH sensitization. **(A)** Fluorescence image showing the location of AAV expression and bilateral NAc (Franklin and Paxinos, 2001) of mouse neurons (arrows). Scale bar, 100 μm. **(B)** Injection of AAV-SYN-shAgo2 into the NAc of mice effectively reduced Ago2 protein and mRNA levels. **(C)** Injection of AAV-SYN-Ago2 into the NAc of mice increased Ago2 protein and mRNA levels. Student’s *t*-test showed ***p < 0.001 compared to the virus control group. The data are presented as the mean ± SEM, *n* = 3–7. **(D)** Ago2 downregulation in NAc neurons enhanced METH sensitization. **(E)** Ago2 overexpression in NAc neurons attenuated METH sensitization. Mixed-measures ANOVA showed the following: *p < 0.05, ***p < 0.001, vs. the corresponding saline groups; &*p* < 0.05, &&*p* < 0.01, vs. AAV-SYN-GFP+METH or AAV-SYN-FLAG+METH; ##*p* < 0.01, compared to the locomotor activities recorded on day 3 within the same group. The data were presented as the mean ± SEM, *n* = 8–12. NAc, nucleus accumbens; METH sensitization, METH-induced locomotor sensitization.

Next, we elucidated whether dysregulation of Ago2 could modulate METH sensitization in mice. Neural-specific AAVs were constructed to over express (AAV-SYN-Ago2) or knockdown (AAV-SYN-shAgo2) Ago2 in neurons and were bilaterally microinjected into the NAc. Neural-specific GFP expression detected in the NAc indicated localized microinjection sites (Figure 2A). The expression of Ago2 in NAc neurons was detected to verify efficient overexpression or downregulation upon microinjection of the respective AAV constructs. The levels of the Ago2 protein (t_(4)_ = 2.786, *p < 0.05) and mRNA (t_(12)_ = 2.637, *p < 0.05) were significantly lower in the NAc of AAV-SYN-shAgo2 mice than in those of AAV-SYN-GFP mice (Figure 2B). Mice microinjected with AAV-SYN-Ago2 showed significant overexpression of the Ago2 protein (t_(4)_ = 3.675, *p < 0.05) and mRNA (t_(14)_ = 4.799, ***p < 0.001) in the NAc (Figure 2C).

After Ago2 knockdown (Figure 2D), all mice showed no significant differences in locomotor activities during the pretest (days 1–2). Mixed-measures ANOVA by Bonferroni’s *post hoc* tests revealed the main effects of AAV (F_(1,28)_ = 4.101, *p* = 0.052), METH (F_(1,28)_ = 681.223, *p* < 0.001) and day (F_(7,22)_ = 106.364, *p* < 0.001) and the interactions of AAV ×day (F_(7,22)_ = 1.603, *p* = 0.187), METH ×day (F_(7,22)_ = 99.744, *p* < 0.001) and AAV ×METH ×day (F_(7,22)_ = 0.592, *p* = 0.756) following Ago2 knockdown. The locomotor sensitization test showed that METH still induced a strong increase in locomotion when METH-treated groups and their corresponding saline-treated groups were compared, regardless of Ago2 knockdown (Figure 2D F_(1, 28)_ = 681.233, ***p < 0.001). Significant METH sensitization was also observed on the challenge day (day 10) compared to day 3 in the same group (##*p* < 0.01). There was no difference between day 7 and day 3 (*p* = 0.176) or day 5 (*p* = 0.659) in the development phase of the AAV-SYN-GFP+METH group. The AAV-SYN-shAgo2+METH group displayed higher locomotor activity than that in the AAV-SYN-GFP+METH group from day 3 to day 6 (F_(1, 28)_ = 5.793, &*p* < 0.05) and even at day 10 (F_(1, 28)_ = 4.578, &*p* < 0.05). There was no significant difference between the AAV-SYN-shAgo2+Saline and AAV-SYN-GFP +Saline groups (day 10, *p* = 0.689).

When Ago2 was overexpressed (Figure 2E), mixed-measures ANOVA by Bonferroni’s *post hoc* tests revealed the effects of AAV (F_(1,44)_ = 11.054, *p* < 0.01), METH (F_(1,44)_ = 693.148, *p* < 0.001), and day (F_(7,38)_ = 129.799, *p* < 0.001), as well as the interactions of AAV ×day (F_(7,38)_ = 2.247, *p* = 0.051), METH ×day (F_(7,38)_ = 119.364, *p* < 0.001) and AAV ×METH ×day (F_(7,38)_ = 1.929, *p* = 0.092). The locomotor sensitization test showed that METH induced a strong increase in locomotion when the METH-treated groups and their corresponding saline-treated groups were compared, regardless of Ago2 overexpression (F_(1, 44)_ = 693.148, ***p < 0.001). Significant METH sensitization was also observed on the challenge day (day 10) compared to day 3 in the same group (###*p* < 0.001). There was no difference between day 7 and day 3 (*p* = 0.129) or day 5 (*p* = 0.974) in the development phase of the AAV-SYN-FLAG+METH group. As expected, locomotion performed by the mice in AAV-SYN-AGO2+METH group was significantly decreased on each METH injection day (day 3, F_(1, 44)_ = 12.886, &&*p* < 0.01) compared to that in the AAV-SYN-FLAG+METH group (Figure 2E). There was also no significant difference between the AAV-SYN-Ago2+Saline and AAV-SYN-FLAG + Saline groups (day 10, *p* = 0.414).

### miR-3068-5p Is an Argonaute2-Dependent miRNA in the Nucleus Accumbens and Can Disrupt Methamphetamine Sensitization

**FIGURE 3:**
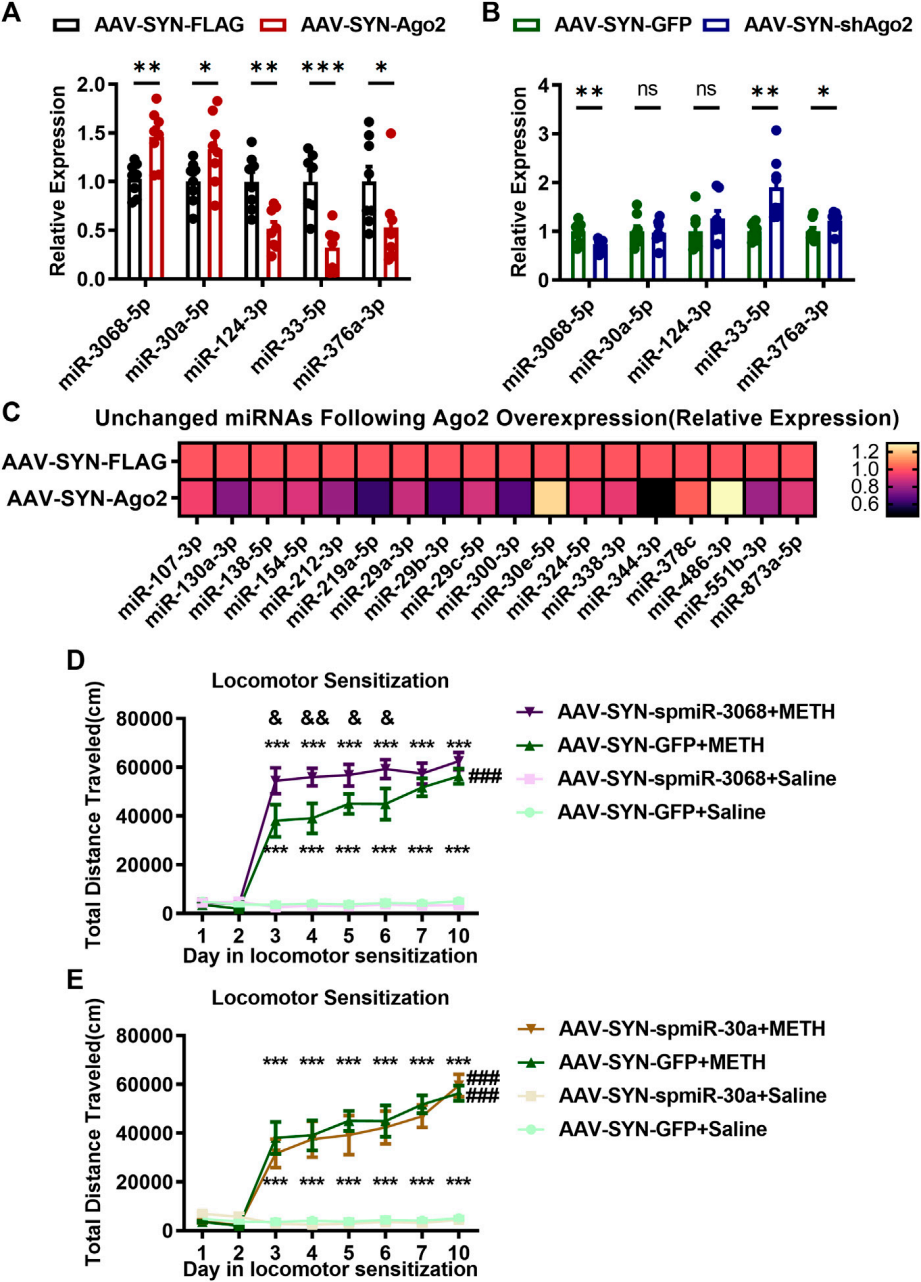
miR-3068-5p was found to be an Ago2-dependent miRNA in the NAc of mice **(A, C)**. Changes in miRNA expression following Ago2 overexpression in the NAc of mice. Student’s *t*-test: *p < 0.05, **p < 0.01, and ***p < 0.001 compared with the AAV-SYN-FLAG group. The data are presented as the mean ± SEM, *n* = 8. **(B)** Changes in miRNA expression in the NAc of mice in the AAV-SYN-shAgo2 group. Student’s *t*-test: *p < 0.05 and **p < 0.01 compared with the AAV-SYN-GFP group. Data are presented as the mean ± SEM, *n* = 8. **(D)** miR-3068-5p interference in NAc neurons strengthens METH sensitization. **(E)** No change in METH sensitization after miR-30a-5p interference in NAc neurons. Mixed-measures ANOVA: ***p < 0.001, vs. the corresponding saline groups; &*p* < 0.05, &&*p* < 0.01, AAV-SYN-spmiR-3068-5p+METH vs. AAV-SYN-GFP+METH; ###*p* < 0.001, compared to the locomotor activities recorded on day 3 within the same group. The data are presented as the mean ± SEM, *n* = 8. NAc, nucleus accumbens; METH sensitization, METH-induced locomotor sensitization.

Considering the miRNA biogenesis role of Ago2, we further verified the Ago2-dependent miRNAs and whether these miRNAs regulated METH sensitization. We detected miRNA expression in the NAc when Ago2 was overexpressed or silenced. Ago2-dependent miRNAs (Schaefer et al., 2010) and miRNAs that were downregulated upon METH treatment (Liu et al., 2019) were selected and verified by qPCR. Interestingly, miR-3068-5p (t_(14)_ = 3.755, **p < 0.01) and miR-30a-5p (t_(14)_ = 2.253, *p < 0.05) were found to be enriched by neuronal Ago2 overexpression in the NAc of mice (Figure 3A). However, we found that three miRNAs (miR-124-3p, miR-33-5p, and miR-376a-3p) were downregulated and 18 miRNAs were unchanged following neuronal Ago2 overexpression (Figure 3C). Surprisingly, miR-3068-5p was the only significantly depleted miRNA (t _(14)_ = 3.117, **p < 0.01) in the NAc following neuronal Ago2 knockdown (Figure 3B). miR-33-5p (t _(14)_ = 3.894, **p < 0.01) and miR-376a-3p (t_(14)_ = 2.203, *p < 0.05) were upregulated in the AAV-SYN-shAgo2 group (Figure 3B), and miR-124-3p and miR-30a-5p were unchanged between the AAV-SYN-GFP and AAV-SYN-shAgo2 groups (Figure 3B). These results indicated that miR-3068-5p may be an Ago2-dependent miRNA in neurons in the NAc.

Therefore, we investigated whether miR-3068-5p also contributes to METH sensitization by intervening with the expression of miR-3068-5p in NAc neurons. A neuron-specific AAV-mediated sponge sequence expression vector for miR-3068-5p (AAV-SYN-spmiR-3068-5p) and the corresponding control vector AAV-SYN-GFP were constructed and microinjected bilaterally into the NAc. The locomotion of mice in response to METH was measured (Figure 3D). Mixed-measures ANOVA by Bonferroni’s *post hoc* tests revealed the effects of AAV (F_(1,28)_ = 4.218, *p* < 0.05), METH (F_(1,28)_ = 291.487, *p* < 0.001), and day (F_(7,22)_ = 73.642, *p* < 0.001), as well as the interactions of AAV ×day (F_(7,22)_ = 3.663, *p* < 0.01), METH ×day (F_(7,22)_ = 75.459, *p* < 0.001), and AAV ×METH ×day (F_(7,22)_ = 1.756, *p* = 0.147). METH still induced a strong increase in locomotion when the METH-treated groups and their corresponding saline-treated groups were compared, regardless of miR-3068-5p inhibition. There was a difference between day 7 and day 3 (*p* = 0.472) or day 5 (*p* = 0.767) in the development phase of the AAV-SYN-spmiR-3068 + METH group, while in the AAV-SYN-GFP+METH group, increased locomotor activity was observed on day 7 compared to day 3 (*p* < 0.01) and day 5 (*p* < 0.01). Significant METH sensitization was observed on the challenge day (day 10) compared to day 3 in the AAV-SYN-GFP group (###*p* < 0.001).

However, miR-3068-5p inhibited METH sensitization on day 10, as observed when day 10 and day 3 in the AAV-SYN-spmiR-3068-5p +METH group were compared (Figure 3D, *p* = 0.065). Interestingly, the AAV-SYN-spmiR-3068-5p +METH group exhibited significant hyperlocomotor activity from day 3 (F_(1, 28)_ = 7.501, &*p* < 0.05) to day 6 (F_(1, 28)_ = 7.141, &*p* < 0.05) compared with the AAV-SYN-GFP +METH group. We also investigated whether miR-30a-5p plays a role in METH sensitization. However, intervening with AAV-SYN-spmiR-30a-5p expression did not change METH sensitization in mice (Figure 3E).

### miR-3068-5p Regulated Methamphetamine Sensitization by Targeting *Grin1*

**FIGURE 4:**
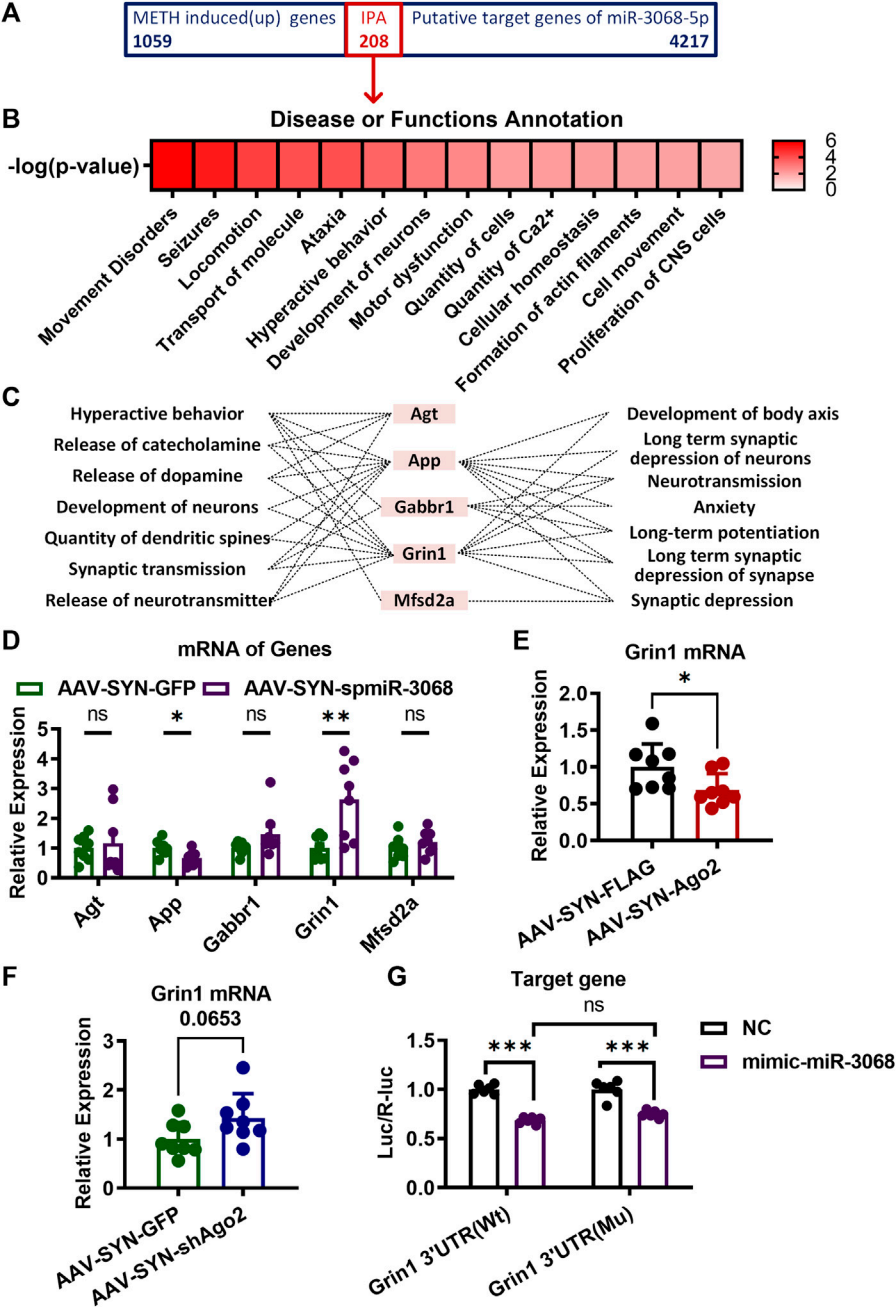
Target genes of miR-3068-5p in NAc of mice involving in METH sensitization. **(A)** A total of 208 overlapping genes between the METH-induced genes in our previous study (https://www.ebi.ac.uk/arrayexpress/, E-MTAB-2843) and the predicted target genes of miR-3068-5p were identified and subjected to IPA analysis. **(B)** Identification of the main neurological dysfunctions of the 208 genes identified from **(A)**. **(C)** IPA analysis identified five overlapping genes from **(A)** relevant to hyperactive behavior, release of catecholamine, release of dopamine, development of neurons, quantity of dendritic spines, synaptic transmission, release of neurotransmitters, development of the body axis, long-term synaptic depression of neurons, neurotransmission, anxiety, long-term potentiation, long-term synaptic depression of synapses, and synaptic depression. **(D)** mRNA expression of the five predicted target genes of miR-3068-5p relevant to locomotion following miR-3068-5p sponging in the NAc of METH-sensitized mice. **(E, F)** Graphs show the expression of *Grin1* mRNA upon overexpression **(E)** or knockdown **(F)** of Ago2. Student’s *t*-test: *p < 0.05 and **p < 0.01 compared to the AAV control group. The data are presented as the mean ± SEM, *n* = 6–8. **(G)** Graphs showing the dual-luciferase activities upon transfection of *Grin1* Wt or mutant (Mu) expression conducted alone (NC) or by cotransfection with mimic-miR-3068-5p. Two-way ANOVA: ***p < 0.001 compared to the corresponding NC group. The data are presented as the mean ± SEM, *n* = 6. NAc, nucleus accumbens; METH sensitization, METH-induced locomotor sensitization; IPA, Ingenuity Pathway Analysis.

Considering the downregulation of miR-3068-5p in response to METH in our previous study (Liu et al., 2019), we speculated that the potential target genes of miR-3068-5p would be upregulated in response to METH. Therefore, comparisons were made between the predicted targets of miR-3068-5p and mRNAs that were upregulated in the NAc of METH-treated mice in our previous study (https://www.ebi.ac.uk/arrayexpress/, E-MTAB-2843), and 208 transcripts were identified as METH-responsive putative targets for miR-3068-5p. These potential targets were further analyzed by IPA to verify their functional characteristics (Figure 4A). Disease and functional annotations classified these targets into several functions, such as hyperactive behavior, release of catecholamine, release of dopamine, development of neurons, quantity of dendritic spines, synaptic transmission, release of neurotransmitters, development of the body axis, long-term synaptic depression of neurons, neurotransmission, anxiety, long-term potentiation, long-term synaptic depression of synapses, and synaptic depression (Figure 4B). Five genes (*Agt, App, Gabbr1, Grin1*, and *Mfsd2a*) were found to be significantly enriched in pathways involved in the regulation of synaptic plasticity, morphology, and development (Figure 4C). We measured the relative expression of *Agt, App, Gabbr1, Grin1*, and *Mfsd2a* in the NAc of AAV-SYN-spmiR-3068-5p mice but did not detect any changes in the expression of *Agt*, *Gabbr1*, or *Mfsd2a* (Figure 4D). Moreover, a significant reduction in *App* was observed in the NAc of mice with AAV-SYN-spmiR-3068-5p microinjection. Only *Grin1* showed significant upregulation (t_(14)_ = 3.408, **p < 0.01) when the expression of miR-3068-5p was disrupted by AAV-SYN-spmiR-3068-5p. We further found that *Grin1* was downregulated (t_(14)_ = 2.306, *p < 0.05) in the NAc when Ago2 was overexpressed (Figure 4E) and showed an upregulation trend (t_(14)_ = 1.999, *p* = 0.065, Figure 4F) in the NAc when Ago2 expression was disrupted (Figure 4F). As such, we used a Dual-Luciferase Reporter assay to further verify whether miR-3068-5p could target *Grin1* expression. As shown in Figure 4G, cotransfection of the *Grin1* 3′UTR (Wt) and mimic-miR-3068-5p resulted in a significant decrease (t_(1, 20)_ = 9.632, ***p < 0.001) in the ratio of the dual-luciferase activities in 293T cells, indicating that miR-3068-5p could indeed inhibit *Grin1* expression. However, this decreased ratio of the dual-luciferase activities in cells was not rescued with *Grin1* 3′UTR (Mu) and mimic-miR-3068-5p cotransfection. The *Grin1* 3′UTR (Mu) alone showed a comparable ratio of the dual-luciferase activities as the *Grin1* 3′UTR (Wt). These data suggest the presence of other atypical binding sites of miR-3068-5p on *Grin1* or other mechanisms in addition to direct targeting of the expression of *Grin1*.

## Discussion

### Argonaute2 in the Nucleus Accumbens Is Important for the Development of Methamphetamine Addiction

Here, we found that Ago2 was progressively downregulated in the NAc of mice during METH sensitization development. We further identified that overexpressing or silencing neural Ago2 could attenuate or enhance METH sensitization, respectively, and especially affect locomotion after the first injection of METH, indicating that Ago2 can regulate the acute response to METH. Evidence has shown that Ago2 is involved in the regulation of neural plasticity. It was reported that Ago2 overexpression can rescue the loss of miRNA activity and decrease dendrite complexity (Störchel et al., 2015). In addition, Ago2 was also found to be involved in changes in NMDAR-modulated dendritic spine morphology (Rajgor et al., 2018). Neddens found that acute METH injection into reared gerbils restrained the development of adult dopamine fiber density in the NAc (Neddens et al., 2002). In our previous study, repeated METH injections led to an increase in synaptic density on medium spiny neurons (MSNs) in the NAc (Zhu et al., 2012). Here, we found repeated METH treatment significantly downregulated Ago2 expression in the NAc of mice and METH-induced sensitization was attenuated when Ago2 was overexpressed in NAc neurons. It is reasonable to assume that Ago2 overexpression attenuated METH sensitization through decreasing synaptic density in the NAc. Thus, further studies are warranted to address whether Ago2 modulates METH addiction by regulating neural transmission in the NAc.

METH is a psychostimulant that induces a hyperlocomotion response by persistently activating dopaminergic transmission in the NAc (Lominac et al., 2014). Kelly et al. (2008) have reported a significant reduction in METH locomotor sensitization of the amplitude and duration in DRD2-deficient mice, regardless of whether they received METH for the first time or after several prior exposures. In addition, Schaefer et al. found that deficiency of Ago2 in DRD2-expressing neurons can result in the downregulation of a subset of miRNAs that may be involved in cocaine motivation (Schaefer et al., 2010). Based on these studies, we speculated that Ago2 expressed in the NAc may be a downstream molecule of DRD2 in response to METH. Since dopamine DRD1- and DRD2-expressing MSNs are the main NAc neurons and play essential roles in drug reward (Lobo et al., 2010) and given that we verified the function of Ago2 expressed in neurons of the NAc in general without separating different dopamine receptor-expressing neurons, the behavioral phenotype that we observed in our study could be a net effect modulated by both types of neurons. Nevertheless, the observed role of Ago2 in cocaine addiction and METH sensitization indicated that Ago2 may play an important role in regulating drug addiction with a neural type-specific pattern.

### miR-3068-5p Could Be a Neural Argonaute2-Dependent Reduced by Methamphetamine (NADRM) miRNA in the Nucleus Accumbens of Mice

Although Dicer1 cleaves miRNA from its precursor to mature form (Knight and Bass, 2001), Ago2 was also found to play a slicer endonuclease role, deficiency of which impaired miRNA biogenesis from precursors to miRNAs and caused a reduction in the expression of miRNAs, such as miR-451 (Cheloufi et al., 2010; Yang et al., 2010). Overexpression or enhanced activity of Ago2 elicited specific changes in miRNAs and mRNAs and had a strong relationship with high-risk myeloma (Zhou et al., 2010; Hagiwara et al., 2012; Zhang et al., 2013). In our previous study (Liu et al., 2019), we found that several METH-reduced miRNAs may be Ago2-dependent. Thus, we determined whether any Ago2-dependent miRNA regulates METH sensitization. Interestingly, we found increased and decreased expression of miR-3068-5p upon overexpression and knockdown of Ago2 in NAc neurons, respectively. miR-3068-5p was one of the potential Ago2-dependent miRNAs found in our previous study (Liu et al., 2019), the downregulated expression of which paralleled the downregulated Ago2 expression, suggesting an involvement of this miRNA in METH sensitization. As expected, decreasing the expression of miR-3068-5p in NAc neurons also enhanced the development of METH sensitization, similar to the behavioral changes mediated by inhibiting Ago2 expression. Although miR-30a-5p was enriched following overexpression of Ago2 in NAc neurons, it was not depleted when Ago2 was downregulated in NAc neurons and did not exhibit any effects on METH sensitization. Collectively, these results suggested that miR-3068-5p could be neural Ago2-dependent reduced by METH (NADRM) miRNA in the NAc of mice. Decreased expression of miR-3068-5p may contribute to METH sensitization.

In the current study, we also observed different alteration patterns of miRNAs upon changes in Ago2 expression. For example, the levels of miR-33-5p and miR-376a-3p were both decreased and increased upon bidirectional regulation of Ago2 expression. There was also a set of miRNAs that were not changed following Ago2 overexpression or knockdown. This phenomenon may be due to the selectivity of Ago2 splicing and other indirect or unknown functions of Ago2 (Yang et al., 2010). Moreover, Ago2 is expressed and functions not only in nerve cells but also in gliocytes (He et al., 2012; Chaudhuri et al., 2018). In addition, reported evidence showed that the PAZ domain of Ago2 was capable of shortening mature miRNA (Juvvuna et al., 2012), which likely explains the opposite expression changes of miR-33-5p and miR-376a-3p following Ago2 regulation.

However, Ago2 is not only involved in specific miRNA biogenesis but also a key component of the RISC involved in miRNA- or siRNA-mediated target mRNA degradation. Thus, the potentially universal effect of Ago2 silencing on mRNA function should be considered. We speculated that the downregulation of Ago2 may induce the hypofunction of RISC and disinhibition of RNAi, which may result in considerable upregulation of mRNA expression. However, in our previous study, mRNAs were greatly downregulated by METH (Zhu et al., 2016). Since mRNA can also be regulated by other transcription factors, the universal changes in mRNA function were neutralized by multiple factors as a complex consequence of the response to METH sensitization. Nevertheless, our study showed that differential expression of Ago2 in the NAc could modulate METH sensitization by regulating miR-3068-5p biogenesis, indicating that Ago2, which plays an important role in miRNA generation and execution of miRNA-mediated gene silencing, is involved in the regulation of METH addiction.

### *Grin1* May Be Involved in the Effects of Argonaute2/miR-3068-5p on Methamphetamine Sensitization

**FIGURE 5:**
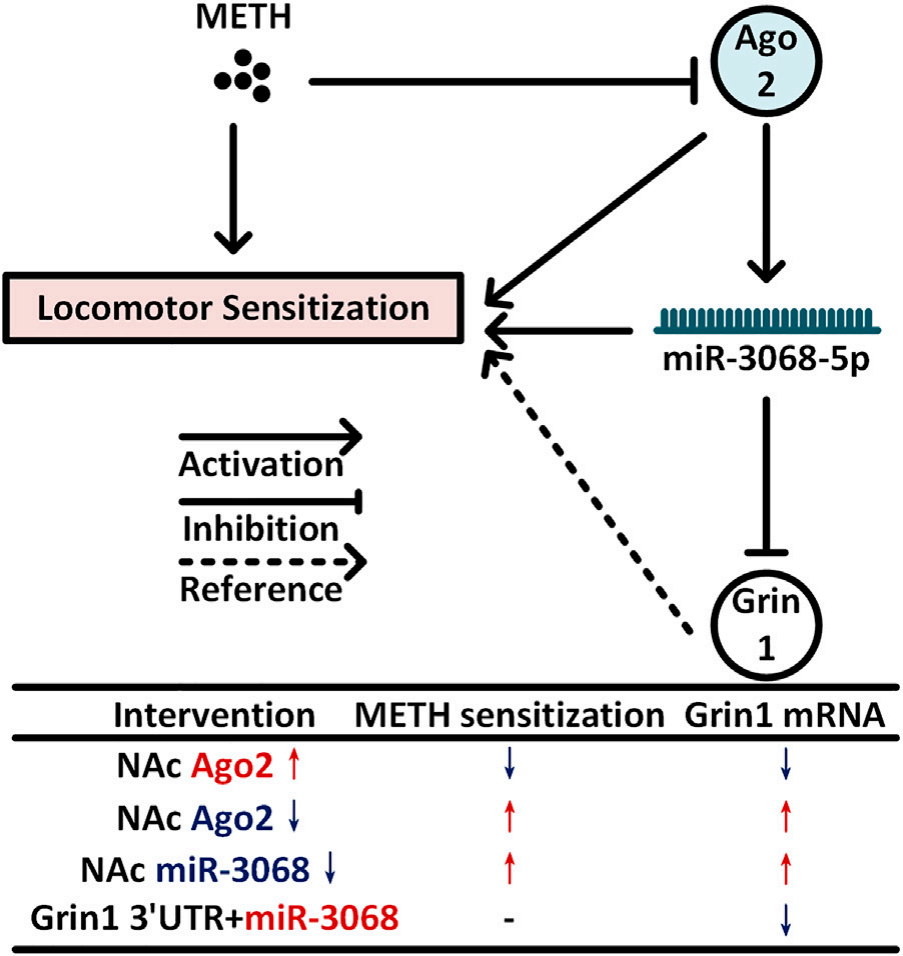
Model for NAc neural Ago2/miR-3068-5p cascades in regulating METH sensitization. METH induces progressive downregulation of Ago2 in the NAc. Downregulation of Ago2 may cause a significant decrease in miR-3068-5p in the NAc and then contribute to METH sensitization. *Grin1*, encoding a critical subunit of NMDAR (Beutler et al., 2011), is a potential downstream gene of miR-3068-5p and may be involved in these effects.

To identify the potential targets of miR-3068-5p in regulating METH sensitization, we predicted the target genes of miR-3068-5p and compared them to the previously identified upregulated genes in the NAc of METH-sensitized mice since miR-3068-5p was downregulated in the NAc of mice in response to METH. We focused on the genes with functions relevant to synaptic plasticity and morphology by IPA, and *Grin1* was the only gene that was upregulated when miR-3068-5p was downregulated by AAV-SYN-spmiR-3068-5p. *Grin1* encodes N-methyl-D-aspartate receptor (NMDAR) subunit 1 (NR1), which is essential to the formation of functioning NMDARs. Specifically, removing NMDAR signaling from DRD1-expressing MSNs could prevent amphetamine sensitization, and this attenuation of sensitization could be rescued by virus-mediated restoration of NR1 (encoded by *Grin1*) in DRD1-expressing neurons in the NAc, demonstrating the requirement of *Grin1* in NAc MSNs for amphetamine sensitization (Beutler et al., 2011). Here, we found that decreasing miR-3068-5p levels in the NAc could enhance METH sensitization and increase *Grin1* expression. More importantly, *Grin1* was downregulated following Ago2 overexpression and showed an upregulation trend when Ago2 was knocked down. These results further indicated that the effects of Ago2/miR-3068-5p on METH sensitization may occur via regulation of *Grin1* in NAc neurons (Figure 5).

## Conclusion and Limitations

In summary, we found that Ago2 was downregulated progressively in the NAc of mice during METH sensitization, and METH sensitization could be attenuated or enhanced by overexpression or knockdown of Ago2 in NAc neurons. Furthermore, miR-3068-5p is considered an NADRM miRNA, and neural Ago2/miR-3068-5p cascades are important for METH sensitization. Downregulation of miR-3068-5p in NAc neurons increased locomotor activity during the development of METH sensitization. This functional role of Ago2/miR-3068-5p is likely to occur through the regulation of *Grin1* in neurons within the NAc (Beutler et al., 2011) (Figure 5).

However, there were also some limitations of this study. First, since the trace of mice in the OF test in the corner partially reflected anxiety behavior, Ago2 overexpression did not change the central area traveled time or distance on the first day when the mice were put into the OF box. Apparently, there was no effect of Ago2 on anxiety-like behavior in this model, but other anxiety tests should be performed in future studies. Second, although Ago2, as a key molecule in RNAi, was found to be widely expressed throughout the brain, there was no evidence showing the expression pattern of miR-3068 in the brain regions. For now, it cannot be determined if the role of Ago2/miR-3068-5p is NAc-specific. In addition, because of the different upstream receptors of Ago2 and different target genes of miR-3068, Ago2/miR-3068-5p may display specific functions in specific neural types, which are needed for further research. Finally, different functions of the NAc core and shell have been reported, but, here, we did not determine the different roles of Ago2/miR-3068 in the NAc core or shell. Considering that Ago2 can modulate the expression of miRNAs in a cell-specific type, the role of Ago2/miR-3068 in the NAc subregion may be different and should be investigated with a deep understanding of neural types, such as DRD1- and DRD2-expressing neurons.

## Data Availability

The original contributions presented in the study are included in the article/Supplementary Material; further inquiries can be directed to the corresponding authors.
