# Supplementary material for: Potential Ago2/miR-3068-5p Cascades in the Nucleus Accumbens Contribute to Methamphetamine-Induced Locomotor Sensitization of Mice
Source: Front Pharmacol. 2021 Aug 13;12:708034. doi: 10.3389/fphar.2021.708034 (PMC8414410; doi:10.3389/fphar.2021.708034)
Supplement: Supplementary file 1 [file Table1.DOC]

**Table 1** qPCR primers

| **Gene** | **Forward (5’-3’)** | **Reverse (5’-3’)** |
| --- | --- | --- |
| *Gapdh* | TGTGTCCGTCGTGGATCTGA | TTGCTGTTGAAGTCGCAGGAG |
| *Dicer1* | GAATTGCTCGAGATGGAACCAGA | AGCTCCGGCCAACACCTTTA |
| *Ago2* | ACATTCCCGCAGGCACAA | GTCATCCCAAAGCACGTGGTAG |
| *Grin1* | GGCTGACTACCCGAATGTCCA | TGTAGACGCGCATCATCTCAAAC |
| *Gabbr1* | ACGTCACCTCGGAAGGTTG | CACAGGCAGGAAATTGATGGC |
| *Msfd2a* | AACAAGCTTTGCTATGCAGTTGGAG | GCTAATGCAGAAGCCCACCAG |
| *Agt* | GGGTCAGTACAGACAGCACCCTA | CGGAGATCATGGGCACAGAC |
| *App* | TTCTGGGCTGACAAACATCAAGAC | GGTGATGACAATCACGGTTGCTA |
